# Corrigendum: Influence of IL28B and MxA gene polymorphisms on HCV clearance in Han Chinese population

**DOI:** 10.1017/S0950268818003321

**Published:** 2019-03-19

**Authors:** Feng Zang, Ming Yue, Yinan Yao, Mei Liu, Haozhi Fan, Yue Feng, Xueshan Xia, Peng Huang, Rongbin Yu

The above article was originally submitted to Epidemiology and Infection with an incorrect funding number. The first funding number from the Natural Science Foundation of Jiangsu Province should have been BK20171054.

This error has now been rectified in the online PDF and HTML version of the article.
